# Nuclear mitochondrial interaction test reveals sex-dependent mitochondrial SNP-Klotho interactions on diabetes risk

**DOI:** 10.1210/jendso/bvag143

**Published:** 2026-06-24

**Authors:** Tae Jung Oh, Hiroshi Kumagai, Kelvin Yen, Eileen M Crimmins, Thalida E Arpawong, Pinchas Cohen

**Affiliations:** Department of Internal Medicine, Seoul National University Bundang Hospital, Seongnam, 13620, Korea; Department of Internal Medicine, Seoul National University College of Medicine, Seoul, 03080, Korea; Leonard Davis School of Gerontology, University of Southern California, Los Angeles, CA 90089, USA; Leonard Davis School of Gerontology, University of Southern California, Los Angeles, CA 90089, USA; Leonard Davis School of Gerontology, University of Southern California, Los Angeles, CA 90089, USA; Leonard Davis School of Gerontology, University of Southern California, Los Angeles, CA 90089, USA; Leonard Davis School of Gerontology, University of Southern California, Los Angeles, CA 90089, USA

**Keywords:** diabetes mellitus, Klotho, mitochondria, single nucleotide polymorphism, sex

## Abstract

**Context:**

Environmental or other genetic factors might influence the effect of *Klotho* (*KL)* on glucose metabolism.

**Objective:**

We investigated mitochondrial genetic variants that interact with *KL* single nucleotide polymorphisms (SNPs) to modulate diabetes risk.

**Methods:**

We used the data from 7047 non-Hispanic White participants of the Health and Retirement Study, a prospective observational study including adults aged 50 years and older from the United States. First, we performed single gene-wide association scans to identify *KL* SNPs associated with diabetes. Next, we performed a nuclear-by-mitochondrial interaction test (NuMIT) in which we use an identified *KL* SNP from the gene-wide scan to evaluate potential interactions with 85 mitochondrial SNPs in relation to diabetes.

**Results:**

We failed to identify a significant association between diabetes and the *KL* SNP in our single gene-wide association test. However, we identified a novel variant (*KL* rs9563121) which showed a trend of increasing Klotho mRNA levels with each additional minor allele. A NuMIT analysis identified mitochondrial SNPs, which showed significant interactions with rs9563121 in relation to diabetes risk. MitoG15929A showed significant interactions with rs9563121 in both men and women. MitoG15929A diminished the potential beneficial effect of *KL* rs9563121 on diabetes risk in women. Among men with the MitoG15929A variant, *KL* rs9563121 was associated with higher prevalence of diabetes.

**Conclusion:**

The NuMIT approach revealed significant interactions between mitochondrial and nuclear DNA variants of *KL*. Furthermore, MitoG15929A may have a role in the interaction between diabetes and *KL* in a sex-dependent manner.

Mice with defects in the *Klotho* gene (*KL*) show effects on age-related phenotypes, such as short lifespans and arteriosclerosis [1], although *KL* overexpression extends lifespans [2]. Therefore, *KL* has been thought to be an aging suppressor. Studies on the human genetic polymorphism of *KL* also suggest anti-aging effects of the Klotho protein in humans [3, 4]. A recent attempt to test the therapeutic potential showed that Klotho protein administration improved memory in old rhesus monkeys [5]. In this context, genetic variations related to *KL* may not only represent a key pathological mechanism of human diseases in conjunction with aging but also have the potential to be therapeutic targets for human chronic diseases.

Type 2 diabetes can develop from imbalances between beta-cell dysfunction and insulin resistance, which are the core pathophysiologic defects of the disease [6]. As aging is related to both beta-cell function and insulin resistance [7, 8], type 2 diabetes is considered an age-related disease. However, the role of Klotho in glucose metabolism is not as simple as with other age-related phenomena such as longevity and cognition. *KL* mutant mice show decreases in insulin production but also increases in insulin sensitivity [9], which implicates action in the opposite direction for systemic glucose levels. Furthermore, overexpression of Klotho in mice pancreatic islets directly increased insulin production [10], while overexpression of *KL* in mice inhibits insulin signaling in vivo [2]. Interestingly, diet can modify the influence of Klotho on glucose metabolism. For example, insulin sensitivity was lower in *KL* transgenic mice and higher in heterozygous mice compared to wild-type mice, respectively. Yet, under high-fat diets, insulin sensitivity was restored in *KL* transgenic mice and decreased in heterozygous mutant mice, a phenomenon that was reversed under the normal chow diet model [11]. Therefore, environmental or other genetic factors, aside from *KL,* could influence the effect of Klotho on glucose metabolism. In this context, more precise evaluation is required to understand the role of Klotho on diabetes. Therefore, we designed this study as a hypothesis-driven candidate gene analysis rather than an exploratory genome-wide association study.

In addition, sex-dimorphism has been observed in the relationship between Klotho and glucose metabolism. For example, insulin resistance was observed exclusively in male transgenic mice and not in their female counterparts [2]. Other human studies also showed sex differences in Klotho levels associated with certain clinical phenotypes of depression [12] and lipid profiles [13]. This complex relationship between Klotho and human diseases prompted us to investigate a novel interaction involving *KL* genetic variations and diabetes considering potential interactions with sex. Furthermore, we included both nuclear and mitochondrial genomic information because most genetic studies analyzed the nuclear genome and mitochondrial genome separately, resulting in interactions between these two potentially being missed.

## Methods

### Study population

The Health and Retirement Study (HRS) is a prospective observational study of community-dwelling adults over 50 years of age from the United States. The first recruitment was conducted in 1992, and the surveyed samples are more than 37 000 individuals. The study participants were followed up biannually. The cohort overview was published elsewhere [14]. In the current study we used the data obtained in 2016. Race was self-reported and grouped as Hispanic, non-Hispanic Black, non-Hispanic White, and other. In this study, we analyzed data from non-Hispanic White individuals (n = 7047) not only because the number of subjects in other ethnic groups was relatively small (non-Hispanic Black n = 1686, Hispanic n = 1288), but also because the allelic frequencies of micohondiral variants differ substantially across ancestries. All participants provided written informed consent. The study was approved by the University of Southern California Institutional Review Board and the HRS cohort study was approved by University of Michigan Health Sciences Institutional Review Board.

### Definition of diabetes, study covariates, and genomic data

Sociodemographic data were obtained by face-to-face interview or telephone. Diabetes was self-reported as diagnosed by a doctor or by the participants. Other variables included age, sex, body mass index (BMI), systolic and diastolic blood pressure, and comorbidities. Genotype data were accessed from the National Center for Biotechnology Information Genotypes and Phenotypes Database (dbGaP) (dbGaP, study accession: phs000428.v2.p2) [15]. Genotyping was conducted on over 15 000 individuals using either the Illumina HumanOmni2.5-4v1 (2006 and 2008) and HumanOmni2.5-8v1 (2010) arrays and was performed by the National Institutes of Health Center for Inherited Disease Research. Standard quality control procedures were implemented by the University of Washington Genetic Coordinating Center. Further detail is provided in the HRS documentation [16]. Mitochondrial single nucleotide polymorphism (SNP) alleles are binary coded as 0 or 1 whereas nuclear SNPs are coded as 0, 1, or 2 for the number of minor alleles.

### mRNA expression levels using RNA-sequencing data

In the HRS cohort, venous blood was collected in PAXgene RNA tubes. Total RNA was extracted with the QIA Cube semi-automated method using the PAXgene Blood miRNA kit. Ribosomal RNA and globin reduction were performed using the TruSeq stranded Globin-Zero Gold rRNA Removal kits. RNA samples were sequenced as 50–base-pair single-read sequences with a minimum of 20 million reads per sample on an Illumina NovaSeq 6000. Sequencing was based on the TopMed/GTEX analysis pipeline, first using the STAR aligner [17] to align RNAseq reads to the GrCh38 reference genome from GENCODE, then calculating quality control metrics using RNASeQC [18]. SAMTools [19] and RSEM [20] were used to obtain gene read counts. Counts were then normalized using the size factor method implemented by the R package DESeq2 [21] to account for sequencing depth differences. Gene expression values were calculated as the log_2_ counts per million (log2cpm). We then extracted the expression value for the *KL* gene.

### Statistical analysis

Descriptive analysis was performed by SPSS version 27.0 (IBM Co., Armonk, NY, USA) and genetic data were analyzed by PLINK. Continuous data are presented as mean and SD and categorical data are presented as counts with percentages. Differences between 2 groups were assessed using Student *t* test or Chi-square test. Analysis of variance (ANOVA) testing was applied for testing the differences of clinical characteristics. Klotho mRNA levels were compared across *KL* rs9563121 genotype groups using ANOVA, stratified by sex. Logistic regression analysis was used to examine the association between genetic variation in the *KL* gene and diabetes status, stratified by sex, because we observed a trend suggesting gene-sex interaction for diabetes. Each model was adjusted for age, BMI, prior stroke, prior angina, and genetic ancestry principal components 1 to 6. These represent the first 6 principal components of genetic ancestry provided by the HRS, derived from principal component analysis to identify population outliers and to adjust for population substructure. Benjamini-Hochberg (BH) false discovery rate (FDR) was calculated to adjust the *P* values for multiple comparisons. Covariates were chosen based on statistical significance and excluding multicollinearity tested by variance inflation factor. The nuclear-by-mitochondrial gene interaction test (NuMIT) was conducted in PLINK 1.9 [22] with the nuclear SNP candidate used to test effect modification of each mitochondrial SNP on diabetes status, by adding a nuclearSNP × mitochondrialSNP interaction term to each model. Sex-specific models were run, adjusting for age, BMI, prior stroke, prior angina, and genetic ancestry principal component 1 to 6. BH FDR was calculated for multiple test correction. Adjusted *P* values less than .05 were considered statistically significant.

## Results

We analyzed 7047 subjects and diabetes prevalence was 26.9% in men and 21.7% in women. Subjects with diabetes were older and had higher BMI, and diabetes-related comorbidities (Table 1).

**Table 1:** Population characteristics stratified by diabetes status in each sex group

	Men			Women		
	Non-Diabetes	Diabetes	*P*	Non-Diabetes	Diabetes	*P*
No. of subjects	2061	759		3308	919	
Age	72.9 (8.7)	73.9 (8.7)	.008	73.1 (10.6)	73.4 (9.9)	.411
BMI (kg/m^2^)	27.6 (4.8)	30.4 (5.5)	<.001	26.9 (5.9)	30.4 (6.7)	<.001
SBP (mmHg)	129 (16)	131 (17)	.031	126 (19)	130 (19)	<.001
DBP (mmHg)	77 (10)	75 (10)	<.001	77 (10)	76 (10)	.768
Comorbidities (no. of case/total, %)						
Stroke	9.8	16.6	<.001	8.8	15.2	<.001
Heart attack	2.1	3.3	.098	1.0	2.7	<.001
Angina	4.4	9.7	<.001	4.0	7.3	<.001
Heart failure	4.1	11.3	<.001	3.8	7.8	<.001

Data are % or mean (SD). *P* values were derived from χ^2^ test or Student *t* test. Abbreviations: BMI, body mass index; DBP, diastolic blood pressure; SBP, systolic blood pressure.

### Association of *KL* SNPs with diabetes risk

**Figure 1:**
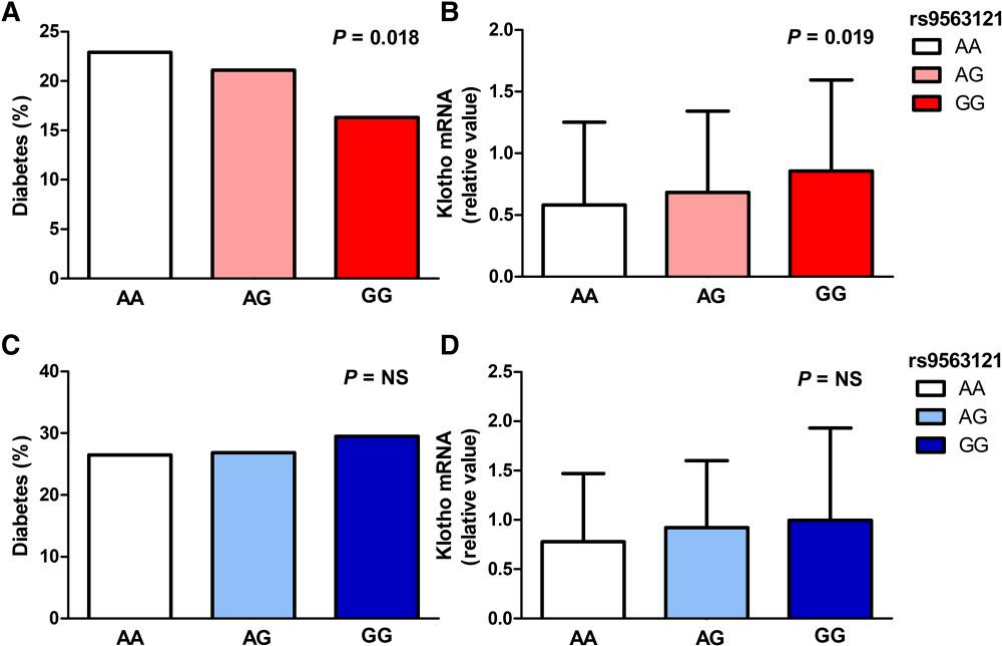
The prevalence of diabetes and Klotho mRNA levels by *KL* rs9563121 genotype. A, prevalence of diabetes in women and B, Klotho mRNA levels in women. C, prevalence of diabetes in men and D, Klotho mRNA levels in men. *P* values are derived from unadjusted Chi-square and ANOVA tests.

First, we performed logistic regression analysis in each sex group (Table 2). In this adjusted model, there were no significant SNPs for diabetes after BH adjustment. However, unadjusted Chi-square test showed a lower prevalence of diabetes among women carrying the minor allele of *KL* rs9563121 compared to women without the minor allele (Fig. 1A). In addition, Klotho mRNA levels were higher in minor allele carriers in women (Fig. 1B). There was no association between diabetes prevalence, *KL* rs9563121 variant and Klotho mRNA in men (Fig. 1C and 1D).

**Table 2:** Logistic regression analysis of the association between KL SNPs and diabetes in each sex group

	Men					Women			
	MA	MAF	OR (95% CI)	*P*	*P* by BH	MAF	OR (95% CI)	*P*	*P* by BH
rs495392	A	0.28	1.19 (1.04-1.36)	.012	0.475	0.29	1.10 (0.97-1.24)	0.126	.576
rs563925*^a^*	A	0.31	1.03 (0.90-1.18)	.681	0.966	0.31	0.86 (0.76-0.97)	0.016	.256
rs568461	G	0.32	0.97 (0.85-1.11)	.633	0.966	0.33	0.91 (0.80-1.02)	0.104	.576
rs571118*^a^*	G	0.45	1.01 (0.89-1.14)	.923	0.972	0.47	0.91 (0.81-1.01)	0.084	.576
rs7982726	G	0.15	0.93 (0.78-1.10)	.389	0.966	0.16	1.07 (0.93-1.25)	0.343	.599
rs9526984	G	0.08	0.94 (0.74-1.20)	.631	0.966	0.08	1.13 (0.92-1.37)	0.243	.596
rs9563121*^a^*	A	0.29	1.02 (0.89-1.17)	.727	0.966	0.29	0.86 (0.76-0.98)	0.018	.256
rs9563124*^a^*	G	0.36	1.03 (0.90-1.17)	.704	0.966	0.37	0.87 (0.77-0.98)	0.018	.256

Logistic regression analysis adjusted for sex, age, BMI, presence of stroke and angina, and eigenvectors 1-6 for genetic ancestry.

BH, Benjamini-Hochberg; MA, minor allele; MAF, MA frequency; OR, odds ratio.

^*a*^Linkage disequilibrium was observed because the D′ value was higher than 0.9.

### Nuclear mitochondrial gene interaction test of *KL* SNP rs9563121

**Figure 2:**
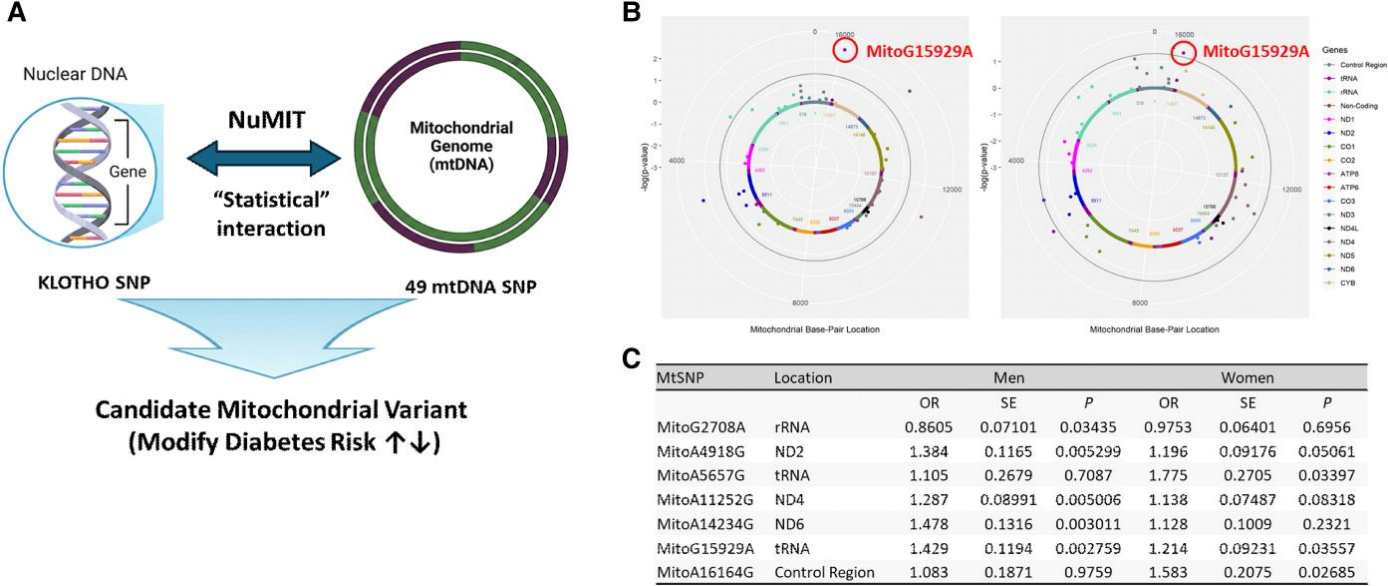
Nuclear-by-mitochondrial gene interaction test (NuMIT): an approach to identify mitochondrial variations that interact with *KL* variant rs9563121 to affect diabetes risk. A, Schematic figure of NuMIT. B, Solar plots presenting results of the NuMIT of *KL* rs9563121 with mitochondrial SNPs. Mitochondrial SNPs extending beyond the outer gray line are statistically significant by a permutation *P* value of .05. Red circles annotate a mitochondrial SNP with a *P* value less than .05 in both men and women. C, Summary of NuMIT between *KL* rs9563121 and mitochondrial SNPs. *P* value was adjusted for age, BMI, prior stroke, prior angina, and genetic ancestry principal components 1-6.

**Figure 3:**
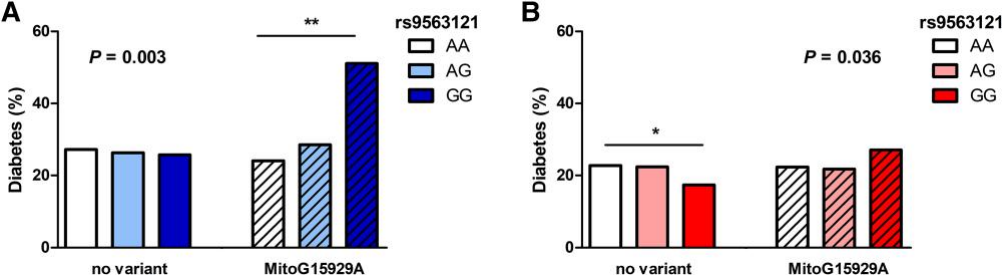
The association between mitochondrial SNP G15929A (MitoG15929A) and diabetes risk according to sex and *KL* SNP rs9563121. A, Men. B, Women. *P* values indicate the interaction between MitoG15929A and rs9563121 on diabetes risk. *P < .05, **P < .01 for Chi-square test.

Given the sex-specific differences in diabetes prevalence and Klotho mRNA observed above, we next tested whether the nuclear SNP of rs9563121 in the *KL* gene significantly interacted with mitochondrial SNPs to be related with risk of diabetes (Fig. 2A). This analysis termed NuMIT identified 7 mitochondrial SNPs that significantly interacted with rs9563121 to be related to diabetes risk. Among them, MitoG15929A significantly modified the association between *KL* rs9563121 and diabetes in men and women (Fig. 2B and 2C). In men with a MitoG15929A minor allele, *KL* rs9563121 was associated with higher prevalence of diabetes (Fig. 3A). In contrast, the beneficial effect of the *KL* rs9563121 minor allele on diabetes was observed in women without MitoG15929A minor allele, but this effect diminished by MitoG15929A (Fig. 3B). However, MitoG15929A itself was not associated with diabetes in either men or women (data not shown).

## Conclusions

Here, we report a novel interaction between a nuclear variant, *KL* SNP rs9563121, and mitochondrial SNP, MitoG15929A. NuMIT can be employed to discover novel genetic interactions within the context of each disease. The *KL* gene and diabetes are one example, and we successfully identified a novel mitochondrial variant through the NuMIT approach. Neither the *KL* SNP nor the mitochondrial SNP showed a significant association with diabetes in the primary analysis based on logistic regression. However, in sex-stratified analyses, differences in diabetes prevalence across KL rs9563121 genotype groups were observed in women.

Previously, genetic variations of *KL* have been reported to interact with another genetic polymorphism. For example, *KL* rs495392 has been reported to interact with *PNPLA3* rs738409, a well-known genetic determinant of fatty liver disease [23]. In that study, the detrimental impact of *PNPLA3* rs738409 on severe hepatic steatosis was attenuated by *KL* rs495392. However, there was no report on nuclear and mitochondrial genetic interactions with *KL* polymorphisms. The genetic interaction between *KL* rs9563121 and MitoG15929A provides an example of how we can better understand nuclear and mitochondrial gene interactions that are associated with diabetes risk. Although the functional role of MitoG15929A has not been fully characterized, previously identified mitochondrial DNA variants are known to influence glucose and insulin metabolism [24]. In addition, mitochondrial-derived microproteins, such as MOTS-c, have been shown to regulate glucose metabolism and insulin sensitivity [25]. Importantly, a genetic variant within the MOTS-c coding region impairs the metabolic effects of MOTS-c, providing evidence that mitochondrial genetic variation can alter peptide function [26]. Further studies are necessary to discover which peptides or molecules derived by this mitochondrial variant can interfere with the effect of Klotho. Furthermore, it is crucial to further investigate the sex-specific effects of rs9563121 and MitoG15929A on glucose metabolism.

Klotho, as an aging suppressor, has been studied for its genetic variations in relation to various chronic diseases. Previous studies showed that rs9563121 was associated with posttraumatic stress disorder [27], while rs571118 and rs563925, which are in linkage disequilibrium with rs9563121, showed delayed onset of end-stage renal disease [28] and low PSA levels [29], respectively. To the best of our knowledge, rs9563121 is reported here for the first time as a genetic variant related to diabetes.

The *Klotho* gene encodes both transmembrane and circulating α-Klotho protein, and the circulating form can function as a hormone [30]. In our study, we did not directly measure the circulating concentration of the Klotho protein. However, we can speculate about the level based on the mRNA expression levels in blood. Interestingly, the protective effect of Klotho was only observed in women. This may partly explain the observed differences in diabetes prevalence according to the presence of minor allele of *KL* rs9563121 in women. Consistent with this observation, the biological effects of Klotho show sex-specific differences in metabolic profiles and clinical outcomes. Klotho transgenic mouse models have demonstrated sex-specific metabolic responses, with a more pronounced increase in insulin resistance in males compared to females [2]. In population-based analysis, lower circulating Klotho levels were associated with adverse metabolic profiles, with stronger associations observed in women, particularly with triglyceride levels [31]. In addition, the relationship between Klotho and mortality appears to differ by sex, with a U-shaped association observed in women but not in men [32]. These sex-specific patterns may be partly explained by differences in sex hormone and fat distribution. For example, an experimental study has shown that Klotho expression can be regulated by estrogen, which contributes to sex differences in biological responses such as stress resilience [33]. Taken together, these findings suggest that the metabolic and prognostic roles of Klotho may differ between sexes. To further understand this sex-dimorphic effect of Klotho on diabetes, additional functional studies incorporating sex hormones will be necessary.

In our study, we have several limitations. First, given the nature of association study, we could not determine the causal effect of Klotho on diabetes. Second, our findings were driven by ethnic-specific phenomena. So, we cannot generalize our findings to other ethnic groups. Third, comorbidities such as stroke and angina were assessed using self-reported physician diagnoses, which may induce misclassification and inconsistency. For example, angina was used as a proxy for ischemic heart disease without distinction from myocardial infarction. Fourth, we did not validate our results using an independent cohort. Lastly, we proposed the interaction between nuclear and mitochondrial genes through statistical methods, but experimental research on actual biology would be necessary to validate this. However, this study has clinical implications, highlighting that consideration of mitochondrial variants in conjunction with nuclear genetic variants is important for assessing chronic disease prevalence.

In conclusion, applying the novel NuMIT approach provided novel insight about the potential interaction between mitochondrial and nuclear DNA effects. MitoG15929A is the first identified mitochondrial variant to modify the risk of diabetes according to the *KL* rs9563121 variant and sex.

## Data Availability

The Health and Retirement Study (HRS) is administered by the University of Michigan. RNA sequencing read counts are currently available to registered users on the HRS website for approved users of Sensitive Health Data (https://hrsdata.isr.umich.edu/data-products/sensitive-health). Additional RNA sequencing data are in the process of being deposited into the NIAGADS Data Sharing Service. Genotyping data for HRS used in this study are available from dbGaP, study accession: phs000428.v2.p2.

## Abbreviations

ANOVA: analysis of variance
BH: Benjamini-Hochberg
BMI: body mass index
dbGaP: Genotypes and Phenotypes Database
FDR: false discovery rate
HRS: Health and Retirement Study
NuMIT: nuclear-by-mitochondrial gene interaction test
SNP: single nucleotide polymorphism

## References

[1] Kuro-o M, Matsumura Y, Aizawa H, et al Mutation of the mouse klotho gene leads to a syndrome resembling ageing. Nature. 1997;390(6655):45‐51. 9363890 10.1038/36285

[2] Kurosu H, Yamamoto M, Clark JD, et al Suppression of aging in mice by the hormone Klotho. Science. 2005;309(5742):1829‐1833. 16123266 10.1126/science.1112766 PMC2536606

[3] Zhu Z, Xia W, Cui Y, et al Klotho gene polymorphisms are associated with healthy aging and longevity: evidence from a meta-analysis. Mech Ageing Dev. 2019;178:33‐40. 30633899 10.1016/j.mad.2018.12.003

[4] Mengel-From J, Soerensen M, Nygaard M, McGue M, Christensen K, Christiansen L. Genetic variants in KLOTHO associate with cognitive function in the oldest old group. J Gerontol A Biol Sci Med Sci. 2016;71(9):1151‐1159. 26405063 10.1093/gerona/glv163 PMC4978356

[5] Castner SA, Gupta S, Wang D, et al Longevity factor klotho enhances cognition in aged nonhuman primates. Nat Aging. 2023;3(8):931‐937. 37400721 10.1038/s43587-023-00441-x PMC10432271

[6] DeFronzo RA . Pathogenesis of type 2 diabetes mellitus. Med Clin North Am. 2004;88(4):787‐835. 15308380 10.1016/j.mcna.2004.04.013

[7] Helman A, Avrahami D, Klochendler A, et al Effects of ageing and senescence on pancreatic beta-cell function. Diabetes Obes Metab. 2016;18(Suppl 1):58‐62. 27615132 10.1111/dom.12719

[8] Spinelli R, Baboota RK, Gogg S, et al Increased cell senescence in human metabolic disorders. J Clin Invest. 2023;133(12):e169922. 37317964 10.1172/JCI169922 PMC10266774

[9] Utsugi T, Ohno T, Ohyama Y, et al Decreased insulin production and increased insulin sensitivity in the klotho mutant mouse, a novel animal model for human aging. Metabolism. 2000;49(9):1118‐1123. 11016890 10.1053/meta.2000.8606

[10] Lin Y, Sun Z. Antiaging gene Klotho enhances glucose-induced insulin secretion by up-regulating plasma membrane levels of TRPV2 in MIN6 beta-cells. Endocrinology. 2012;153(7):3029‐3039. 22597535 10.1210/en.2012-1091 PMC3380305

[11] Gu H, Jiang W, You N, et al Soluble Klotho improves hepatic glucose and lipid homeostasis in type 2 diabetes. Mol Ther Methods Clin Dev. 2020;18:811‐823. 32953932 10.1016/j.omtm.2020.08.002 PMC7479259

[12] Zhang Y, Lu J, Huang S, Chen Y, Fang Q, Cao Y. Sex differences in the association between serum alpha-Klotho and depression in middle-aged and elderly individuals: a cross-sectional study from NHANES 2007-2016. J Affect Disord. 2023;337:186‐194. 37236270 10.1016/j.jad.2023.05.073

[13] Lee J, Kim D, Lee HJ, Choi JY, Min JY, Min KB. Association between serum klotho levels and cardiovascular disease risk factors in older adults. BMC Cardiovasc Disord. 2022;22(1):442. 36221064 10.1186/s12872-022-02885-2 PMC9552482

[14] Sonnega A, Faul JD, Ofstedal MB, Langa KM, Phillips JW, Weir DR. Cohort profile: the health and retirement study (HRS). Int J Epidemiol. 2014;43(2):576‐585. 24671021 10.1093/ije/dyu067 PMC3997380

[15] dbGaP . Health and Retirement Study. National Center for Biotechnology Information: Bethesda, MD. 2012.

[16] HRS . Quality control report for genotypic data. University of Washington. St. Louis, MO. 2012:44.

[17] Dobin A, Davis CA, Schlesinger F, et al STAR: ultrafast universal RNA-Seq aligner. Bioinformatics. 2013;29(1):15‐21. 23104886 10.1093/bioinformatics/bts635 PMC3530905

[18] DeLuca DS, Levin JZ, Sivachenko A, et al RNA-SeQC: RNA-Seq metrics for quality control and process optimization. Bioinformatics. 2012;28(11):1530‐1532. 22539670 10.1093/bioinformatics/bts196 PMC3356847

[19] Li H, Handsaker B, Wysoker A, et al Genome project data processing S. The sequence alignment/map format and SAMtools. Bioinformatics. 2009;25(16):2078‐2079. 19505943 10.1093/bioinformatics/btp352 PMC2723002

[20] Li B, Dewey CN. RSEM: accurate transcript quantification from RNA-Seq data with or without a reference genome. BMC Bioinformatics. 2011;12(1):323. 21816040 10.1186/1471-2105-12-323 PMC3163565

[21] Love MI, Huber W, Anders S. Moderated estimation of fold change and dispersion for RNA-Seq data with DESeq2. Genome Biol. 2014;15(12):550. 25516281 10.1186/s13059-014-0550-8 PMC4302049

[22] Chang CC, Chow CC, Tellier LC, Vattikuti S, Purcell SM, Lee JJ. Second-generation PLINK: rising to the challenge of larger and richer datasets. Gigascience. 2015;4(1):7. 25722852 10.1186/s13742-015-0047-8 PMC4342193

[23] Liu WY, Zhang X, Li G, et al Protective association of Klotho rs495392 gene polymorphism against hepatic steatosis in non-alcoholic fatty liver disease patients. Clin Mol Hepatol. 2022;28(2):183‐195. 34839623 10.3350/cmh.2021.0301 PMC9013609

[24] Kraja AT, Liu C, Fetterman JL, et al Associations of mitochondrial and nuclear mitochondrial variants and genes with seven metabolic traits. Am J Hum Genet. 2019;104(1):112‐138. 30595373 10.1016/j.ajhg.2018.12.001 PMC6323610

[25] Lee C, Zeng J, Drew BG, et al The mitochondrial-derived peptide MOTS-c promotes metabolic homeostasis and reduces obesity and insulin resistance. Cell Metab. 2015;21(3):443‐454. 25738459 10.1016/j.cmet.2015.02.009 PMC4350682

[26] Kumagai H, Miller B, Kim SJ, et al Novel insights into mitochondrial DNA: mitochondrial microproteins and mtDNA variants modulate athletic performance and age-related diseases. Genes (Basel). 2023;14(2):286. 36833212 10.3390/genes14020286 PMC9956216

[27] Wolf EJ, Morrison FG, Sullivan DR, et al The goddess who spins the thread of life: Klotho, psychiatric stress, and accelerated aging. Brain Behav Immun. 2019;80:193‐203. 30872092 10.1016/j.bbi.2019.03.007 PMC6660403

[28] Bostrom MA, Hicks PJ, Lu L, Langefeld CD, Freedman BI, Bowden DW. Association of polymorphisms in the klotho gene with severity of non-diabetic ESRD in African Americans. Nephrol Dial Transplant. 2010;25(10):3348‐3355. 20466664 10.1093/ndt/gfq214 PMC2948839

[29] Kim HJ, Lee J, Lee SY, et al The association between KL polymorphism and prostate cancer risk in Korean patients. Mol Biol Rep. 2014;41(11):7595‐7606. 25120167 10.1007/s11033-014-3647-y

[30] Landry T, Shookster D, Huang H. Circulating alpha-klotho regulates metabolism via distinct central and peripheral mechanisms. Metabolism. 2021;121:154819. 34153302 10.1016/j.metabol.2021.154819 PMC8277751

[31] Wang Z, Zhang H, Zheng G, Wang Z, Shi L. Gender-specific association between circulating serum Klotho and metabolic components in adults. BMC Endocr Disord. 2024;24(1):198. 39334012 10.1186/s12902-024-01737-8 PMC11430003

[32] Wang J, Bai L, Ye Y, Chen X, Hu X, Peng Y. Sex differences in mortality risk and U-shaped relationship with klotho levels: a long-term cohort study. Exp Gerontol. 2024;198:112643. 39613274 10.1016/j.exger.2024.112643

[33] Tan Z, Li Y, Guan Y, et al Klotho regulated by estrogen plays a key role in sex differences in stress resilience in rats. Int J Mol Sci. 2023;24(2):1206. 36674721 10.3390/ijms24021206 PMC9862442

